# Carbon Dots Embedded Hybrid Microgel with Phenylboronic Acid as Monomer for Fluorescent Glucose Sensing and Glucose-Triggered Insulin Release at Physiological pH

**DOI:** 10.3390/nano12173065

**Published:** 2022-09-03

**Authors:** Jinhua Zhu, Wei Liu, Bowen Zhang, Danyang Zhou, Xiangze Fan, Xiaoge Wang, Xiuhua Liu

**Affiliations:** Henan International Joint Laboratory of Medicinal Plants Utilization, College of Chemistry and Chemical Engineering, Henan University, Kaifeng 475004, China

**Keywords:** hybrid microgel, insulin, drug delivery, diabetes

## Abstract

A multifunctional and biocompatible hybrid microgel (poly(VPBA-AAm)-CD) using N, S-doped carbon dots (CDs) and ethylene glycol dimethacrylate (EGDMA) as cross-linking agents, and 4-vinylbenzene boronic acid (VPBA) and acrylamide (AAm) as monomers, was designed in this work. This microgel can be easily prepared by a simple one-pot radical dispersion polymerization of the reactants using a rationally designed hydrogen-bonded complex method. The hybrid microgels were spherical particles with a smooth surface and an average particle size of 234 ± 8 nm. The poly(VPBA-AAm)-CD microgel displayed the glucose-responsive swelling within a clinically concerned range at a physiological pH and could realize the controllable release of insulin. In addition, the release rate of insulin in the hybrid microgel (poly(VPBA-AAm)-CD) could be triggered by glucose concentrations in the solution, and the increasing glucose concentrations can accelerate the insulin release. Further in vitro cytotoxicity studies showed that the microgel had good biocompatibility and no obvious toxicity to the cells. These indicate that the prepared microgel (poly(VPBA-AAm)-CD) may supply a new pattern for the self-regulating therapy of insulin deficiency in diabetes.

## 1. Introduction

With the change in people’s lifestyle and the aging population, diabetes has become a common chronic disease endangering human health [1]. According to the statistics of the International Diabetes Federation (IDF), in 2021, approximately 537 million adults worldwide have diabetes. It is estimated that by 2040, this figure will rise to 642 million and 783 million by 2045 [2]. Therefore, the prevention and treatment of diabetes have been increasingly paid attention to all over the world. At present, there is still no good way to cure diabetes, and the periodical monitoring of glucose concentrations and subcutaneous injections of human insulin remain the main method of controlling insulin-dependent diabetic patients in clinics [3,4]. However, this means of administration may cause pain and infection at the injection site, which can easily result in psychological tension among patients with diabetes [5]. Moreover, it is also important to precisely control the administration dosage of insulin, as high or low doses may easily cause hypoglycemia or hyperglycemia [6,7]. Therefore, it is of great significance that a new type of insulin delivery system is developed to reduce the injection frequency and realize the controlled release of insulin according to the rapid change of blood glucose [8,9].

Glucose-responsive systems have been widely investigated in the field of insulin delivery which can control drug release with the fluctuation of blood glucose. Volpatti et al. [10] developed a glucose-responsive delivery system by coupling glucose oxidase with a stimuli-responsive biomaterial. This microgel had a rapid glucose response, and the blood glucose of diabetes mice was normal for more than 3 weeks after taking the drug carrier reagent. However, the protein-based detection system has certain disadvantages—its high cost, low stability, poor heat resistance and being difficult to use under non-physiological conditions [11]. Materials containing phenylboronic acids (PBAs) have been extensively used as glucose-responsive materials for insulin delivery due to their ability to form dynamic borates with diol-containing molecules [12,13,14]. Compared with protein-based systems, a PBA-based system manifests more stable features under non-physiological conditions and will not cause adverse immune reactions. Hence, in recent years, functional PBA groups are usually incorporated and connected to the polymer crosslinking network to endow the system with glucose-sensitive properties for insulin delivery. Cui et al. synthesized core–shell nanoparticles by the self-assembly of a phenylboronic acid-based block copolymer and a fluorescent complex based on cross-linking between the phenylboronic acid- and glucosamine-containing blocks [15]. The nanoparticles can be tuned via thermo-induced collapse or glucose-induced swelling at an appropriate pH and temperatures and would have potential applications in glucose-responsive drug delivery for diabetes treatment. Holz et al. prepared a glucose-responsive boronic acid-functionalized polymer using a hyaluronic acid (HA) polymer backbone modified with 3-aminophenylboronic acid [16]. Furthermore, the hydrogel was capable of self-healing after physical rupture and had potential utility for sustained drug-release applications. In order to understand the interaction of immobilized PBAs with glucose in hydrogel systems under physiological conditions, Zhang et al. investigated the glucose-dependent volumetric changes of a series of hydrogel sensors functionalized with different classes of PBAs [17]. They found that the volumetric change of the PBA-modified hydrogels was highly dependent on the chemical structure of the immobilized PBAs. Hence, PBAs with specific desired properties can be designed according to the findings of their report.

A microgel is a soft and deformable colloidal particle with a nano-sized network structure which can respond to the expansion or contraction of external stimulated solvents in a short time [18]. Additionally, it has been used in sensors, transport carriers, lotion stabilization, microreactors, etc. [19,20]. Carbon dots (CDs) are dispersed spherical carbon particles with a dimension of less than 10 nm that possess excellent fluorescence, photochemical and chemiluminescent properties [21]. These have been widely used in biosensors [22], bioimaging [23], drug loading [24], etc., due to their high chemical and photochemical stability, low toxicity, lack of photobleaching and absence of blinking [25,26,27,28]. Therefore, after being functionalized with CDs and therapeutic agents, boronic acid-based hybrid microgels can monitor the optical signal change as a function of glucose concentration [29,30,31,32,33,34,35]. Such responsive polymeric microgels are expected to be able to sense the glucose level in the human body, and then respond by releasing a suitable amount of insulin according to the needs of diabetic patients [36,37,38,39,40].

In this work, we prepared a multifunctional hybrid microgel poly(AAm-VPBA)-CD through one-pot radical polymerization using hydroxyl and carboxyl functionalized carbon dots (CDs) and ethylene glycol dimethacrylate (EGDMA) as crosslinking agents, 4-vinyl phenylboronic acid (VPBA) and acrylamide (AAm) as monomers. The boric acid-based microgel with CDs as fluorescent probes showed excellent fluorescent glucose recognition and can be used as a carrier for glucose-responsive insulin delivery. In addition, the results of the cytotoxicity test demonstrated that poly(Aam-VPBA)-CD had a good application prospect in the biomedical field.

## 2. Materials and Methods

### 2.1. Materials and Apparatus

All the chemicals used were of analytical grade. Insulin (porcine pancreas) was purchased from Shanghai Yuanye Bio-Technology Co., Ltd. (Shanghai, China). L-tryptophan, glutathione, 4-vinylphenyl boronic acid, 2,2’-azodiisobutyronitrile (AIBN), EGDMA, acrylamide, sodium dodecyl sulfate (SDS) and BCA protein quantitative kit were acquired from Shanghai Aladdin Biochemical Technology Co., Ltd. (Shanghai, China). Sodium hydroxide, hydrochloric acid (HCl), potassium chloride and dopamine hydrochloride were bought from Tianjin deen Chemical Reagent Co., Ltd. (Tianjin, China). The reagents and materials used in the cell experiments were bought from Zhengzhou Purcell Life Technology Co., Ltd. (Zhengzhou, China). The human normal liver (HL-7702) (Procell CL-0111) cell lines were kindly provided by Procell Life Science & Technology Co., Ltd (Wuhan, China). Ultra-pure water was used throughout.

Fourier transform infrared spectroscopy (FTIR) spectra were recorded between 4000 and 400 cm^−1^ using a VERTEX 70 (Brooke, Germany) Spectrometer. The lyophilized sample was pressed together with potassium bromide to prepare the test discs at room temperature (20 °C). The fluorescence tests were performed on an FLS920 fluorescence spectrophotometer (Edinburgh, UK). The lyophilized microgel was resuspended in PBS (pH 7.4) to measure the particle size using a Malvern Zetasizer Nano ZS90 (Malvern, Worcestershire, UK) at a detection angle of 90 ° at 20 °C. The measurements were based on three repeated results for each sample. The scanning electron microscopy (SEM) figures were acquired using a LEO 1530 field emission SEM (Carl Zeiss NTS GmbH, Oberkochen, Germany). Two sample preparation methods were adopted for SEM images: freeze-dried samples were glued on conductive adhesive; and the ethanol dispersed freeze-dried sample was dropped onto a silicon wafer (for Energy Dispersive X-Ray spectrum). Transmission electron microscopy (TEM) pictures were taken with a JEOLJEM-2010 microscope (Tokyo, Japan) (accelerating voltage was 200 kV). Samples were prepared by dropping an ultrasound-dispersed ethanol solution of lyophilized microgels onto a copper net. The thermal stability of lyophilized samples was measured using a thermogravimetric analyzer (TGA, TGA/DSC3+, METTLER TOLEDO, Zurich, Switzerland). The samples were heated from 25 °C to 800 °C at a rate of 10 °C/min. MTT (3-[4,5-dimethylthiazol-2-yl]-2,5 diphenyl tetrazolium bromide) assays were carried out on a CLARIO star microplate reader (BMG LABTECH, Ortenberg, Germany).

### 2.2. Synthesis of Poly(VPBA-AAm)-CD Hybrid Microgel

The synthesis of CDs was the same as depicted in our previous work [41]. Briefly, glutathione (0.3010 g) and L-tryptophan (0.1022 g) were dissolved in 10 mL of ultrapure water. The mixed solution was then transferred to a high-pressure kettle (30 mL) lined with Teflon and heated at 200 °C for 8 h. Then, the reactor was automatically cooled to room temperature. The solution was centrifuged at 16,000 r/min for 5 min, the precipitate was washed with ultrapure water three times and the washing solution was combined with the supernatant to obtain black solid CDs by freeze-drying. The lyophilized CDs were used to prepare solutions with different concentrations for subsequent poly(VPBA-AAm)-CD hybrid microgels synthesis.

Poly(VPBA-AAm)-CD hybrid microgels were synthesized as follows: 100 mL of the above CDs aqueous solution (0.1 mg/mL) and AAm (0.1020 g) were mixed in a 250 mL round bottom flask, heated to 65 °C and then stirred for 30 min. Subsequently, VPBA (1.84 g), EGDMA (96.4 µL) and SDS (0.0508 g) were added to the above mixed solution with agitation. The polymerization was initiated by adding 0.105 M AIBN (0.064 g) solution after continuous heating in the presence of N_2_ for 60 min. The polymerization was carried out for 6 h. Then, the product was centrifuged at 16,000 rpm for 30 min, the upper layer was discarded and the precipitate was redistributed in 100 mL of ultrapure water. This centrifugation–dispersion process was repeated three times. In order to eliminate free CDs and unreacted monomers, 100 mL of the obtained mixed microgel was dialyzed (8~14 KDa) at room temperature for 10 days. During this period, the water outside the dialysis bag was frequently changed and thus the microgel was further purified [7]. The final purified solution was preserved as the stock solution for hybrid microgels. Solid poly(VPBA-AAm)-CD hybrid microgels can be obtained by freeze-drying the stock solution. Poly(VPBA-AAm) microgels were prepared following the same procedure without CDs.

### 2.3. Fluorescence Deternination Procedure

The fluorescence emission spectra were recorded at 375 nm. The slit widths of emission and excitation were both 1 nm. The fluorescence response of poly(VPBA-AAm)-CD hybrid microgels to glucose was carried out according to the following procedure. A total of 300 µL of the above poly(VPBA-AAm)-CD hybrid microgels stock solution was diluted to 3 mL with PBS (pH 7.4), and then different volumes of glucose were added to investigate the fluorescence variation of the system; fluorescence emission spectra were recorded after 5 min of incubation at room temperature. When the fluorescence characteristics of the poly(VPBA-AAm)-CD were investigated, the original stock solution was diluted with ultrapure water, and the other conditions were the same. When the selectivity of poly(VPBA-AAm)-CD to metal ions was investigated, the volume of the stock solution was changed to 30 µL, and other conditions were unchanged. Each sample was analyzed in triplicate.

### 2.4. Insulin Loading on Poly(VPBA-AAm)-CD Microgels

The insulin solution was prepared with 5.0 mM (pH 7.4) PBS to give a concentration of 0.5 mg/mL and stored in a refrigerator at 4 °C for standby. The lyophilized hybrid microgel was dispersed with PBS (5.0 mM, pH 7.4) to prepare a 0.5 mg/mL solution. Twenty-five milliliters of the hybrid microgel dispersion was stirred in an ice water bath for 12 h, and then twenty-five milliliters of the above prepared insulin solution was dropped into the microgel solution and stirred for 12 h. The reaction solution was then centrifuged at 12,000 rpm for 30 min, and the precipitate was freeze-dried to obtain insulin-loaded hybrid microgels. The supernatant was collected to measure the content of free insulin using the bicinchoninic acid (BCA) protein assay. The amount of insulin loaded on the hybrid microgel was calculated by the difference between the initial mass of insulin added and the amount of unloaded insulin remaining in the supernatant. The drug loading efficiency (DL) and encapsulation efficiency (EE) were used to evaluate the loading capacity [42]. The EE and DL for insulin were calculated using the following formulas:

(1) $$\mathrm{DL}\;\left(\%\right)=\frac{W_{\mathrm{total}\;}-W_{\mathrm{free}}}{W_{\mathrm{poly}\left(\mathrm{VPBA}-\mathrm{AAm}\right)–\mathrm{CD}}}\times 100\%$$

(2) $$\mathrm{EE}\;\left(\%\right)=\frac{W_{\mathrm{total}\;\mathrm{loaded}}-W_{\mathrm{unencapsulated}}}{W_{\mathrm{total}\;\mathrm{loaded}\;}}\times 100\%$$

where W_total_ is the amount of added insulin (mg), W_free_ is the free insulin remaining in the supernatant (mg), W_total loaded_ is the amount of insulin loaded onto the hybrid microgels (mg, W_total loaded_ = W_total_ − W_free_) and W_unencapsulated_ is the amount of free unencapsulated insulin in the hybrid microgels (mg) determined by ultrafiltration centrifugation (10 KDa) method. Each sample was analyzed in triplicate.

### 2.5. Insulin Release In Vitro under Glucose-Controlled Conditions

Five milligrams of the hybrid microgel loaded with insulin was redistributed into 2.5 mL of PBS solution (5.0 mM, pH 7.4) with different concentrations of glucose (0 mM, 5 mM, 10 mM and 20 mM). The solution was rotated and vibrated at 100 rpm at 37 °C and sampled at scheduled time intervals to determine the amount of insulin released. During the sampling, the sample solution was centrifuged at 6000 rpm for 5 min, and 0.2 mL of the supernatant was taken away and an isometric fresh PBS with a corresponding concentration of glucose was replenished for the following tests. The content of free insulin was measured by BCA protein assay. The cumulative release efficiency of insulin molecules in the hybrid microgel of poly(VPBA-AAm)-CD was calculated as follows:

(3) $$\mathrm{Cumulative}\;\mathrm{release}\;\left(\%\right)=\frac{C_{n}{\times V}_{0}{+V}_{i}\sum_{i=1}^{n-1}C_{i}}{m}\times 100$$

where *C_n_* (µg/mL) is the concentration of insulin sampled at a given time. *V*_0_ and *V_i_* (mL) represent the total volume of the release medium and the sampling volume, respectively, and *m* (mg) refers to the mass of insulin in microgels. Each sample was analyzed in triplicate.

### 2.6. Cytotoxicity Assay

The in vitro cytotoxicity research carried out in this study was approved by the Ethics Committee of Biomedical Scientific Research of Henan University (HUSOM2020-037) on 20 March 2020. HL-7702 cell lines were maintained in DMEM (Dulbecco’s modified Eagle’s medium) supplemented with 10% (*v*/*v*) FBS (fetal bovine serum), 100 U/mL penicillin and 100 U/mL streptomycin under an atmosphere of humidified 5% CO_2_ at 37 °C. Cells were dissociated by trypsinization and then passaged when they reached a density of 10^6^ cell/mL.

Poly(VPBA-AAm)-CD microgels on cell viabilities were researched by MTT assay in this study. HL-7702 cells in logarithmic growth phase were incubated in 96-well plates at a density of 3 × 10^3^ cells per well, after incubation for 24 h at 5% CO_2,_ and 37 °C. Subsequently, 200 μL of different concentrations (0, 100, 200, 400 µg/mL) of poly(VPBA-AAm)-CD microgel solutions were added to replace the culture medium, followed by incubation for another 24 and 48 h, respectively. Then, 200 µL (0.5 mg/mL) of MTT was added, and the cells were cultured for 4 h. Subsequently, 150 µL of DMSO (dimethyl sulfoxide) was introduced to each well after the meticulous elimination of the supernatants, and then the 96-well plate was placed on the oscillator and gently shaken to fully dissolve the crystallized formazan in DMSO. Finally, the absorbance of the solution in each well was measured at 490 nm with a microplate reader.

### 2.7. Statistical Analysis

Statistical analysis was analyzed by one-way ANOVA through SPSS 17.0 software (SPSS, version 17.0; Software For Statistical Product Service Solutions; SPSS and IBM Research: Chicago, USA, 2008), and all data are expressed as mean ± SD. Meanwhile, the significant differences between groups were evaluated by Tukey post hoc test, and *p* < 0.05 was considered statistically significant.

## 3. Results

### 3.1. Structure Characterization

**Figure 1:**
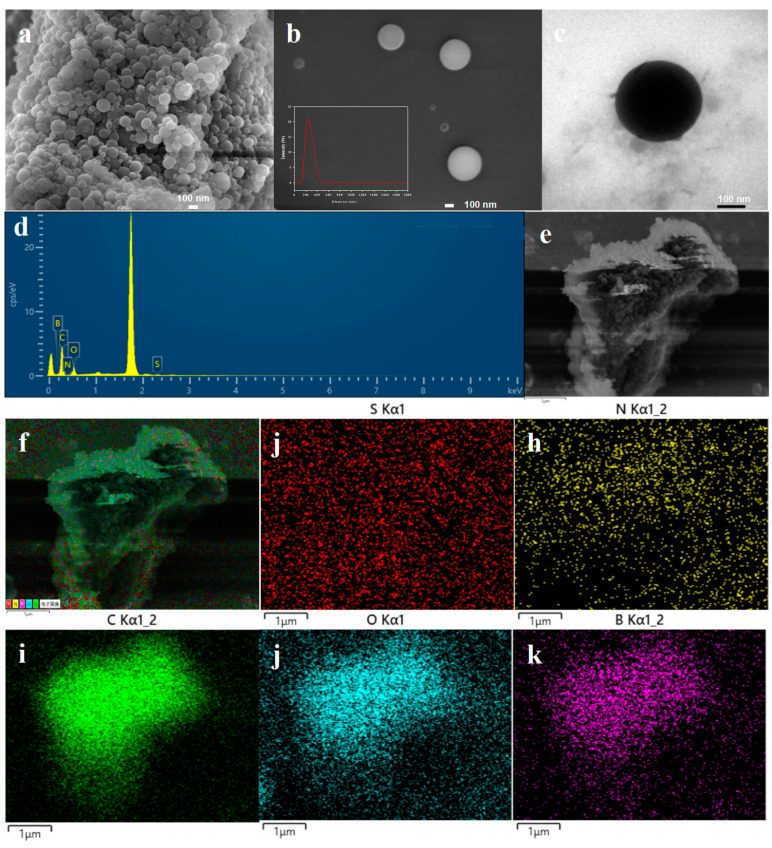
SEM (**a**,**b**), TEM (**c**), X-ray energy spectra (**d**) and elemental distribution (**e**–**k**) of poly(VPBA-AAm)-CD. The inset in b is the particle size distribution of poly(VPBA-AAm)-CD hybrid microgel.

The scanning electron microscope (SEM) and transmission electron microscope (TEM) images of the hybrid microgel poly(VPBA-AAm)-CD are displayed in Figure 1a–c. Obviously, the hybrid microgel of poly(VPBA-AAm)-CD had a smooth spherical morphology. The particle size was mainly distributed between 100 and 400 nm, whilst the average particle size was 234 ± 8 nm (inset of Figure 1b). The microstructure and composition distribution of the poly(VPBA-AAm)-CD hybrid microgel were specifically studied by SEM coupled with energy-dispersive X-ray spectroscopy (EDX). Figure 1f shows the superimposed EDX diagrams of C, O, S, N and B. The overlayed EDX map indicates that C was the main element of the microgels, and its ambiences were crosslinked with N, S, O and B. The mass percentages of O, C, S, N and B in the poly(VPBA-AAm)-CD hybrid microgel were calculated to be 23.98%, 69.06%, 1.31%, 0.38% and 5.28%, respectively (Figure 1d).

**Figure 2:**
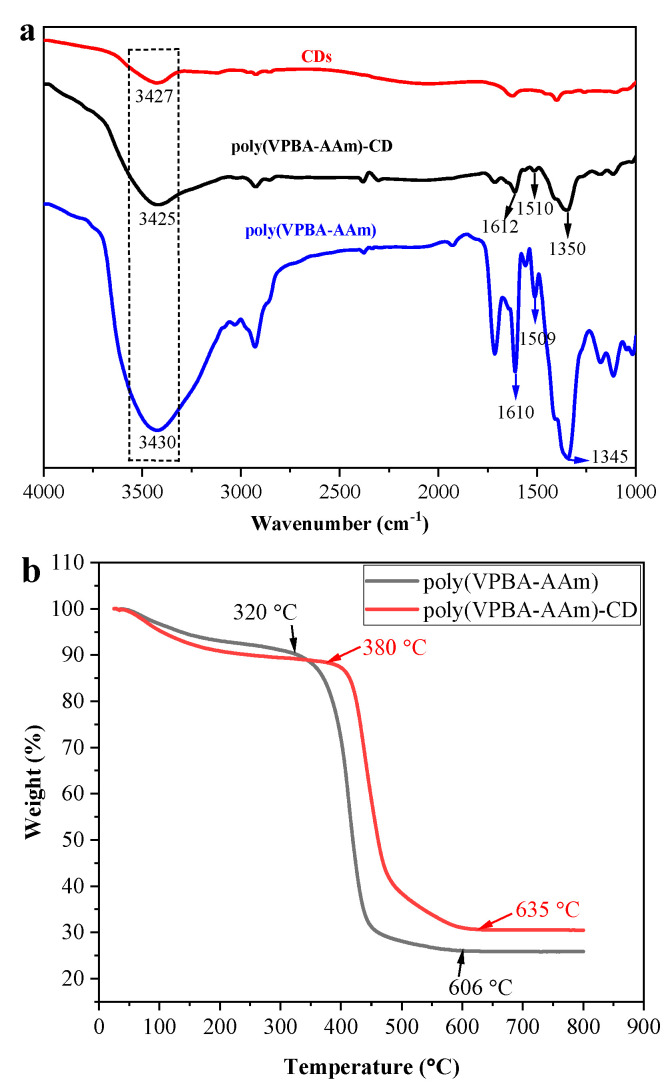
(**a**) FT-IR spectra of free CDs, poly(VPBA-AAm) microgel and poly(VPBA-AAm)-CD hybrid microgel; and (**b**) TGA curves of poly(VPBA-AAm) and poly(VPBA-AAm)-CD microgels.

The FTIR spectra of poly(VPBA-AAm)-CD, free CDs and poly(VPBA-AAm) are shown in Figure 2a. The characteristic absorption peak of hydroxyl on the surface of CDs appear in the spectrum of poly(VPBA-AAm)-CD hybrid microgel, however, the corresponding band shifted from 3427 cm^−1^ to 3425 cm^−1^ [43]. Additionally, the peaks for amide I band at 1509 cm^−1^ and amide II band at 1610 cm^−1^ in poly(VPBA-AAm) were presented in the spectrum of the hybrid microgel of poly(VPBA-AAm)-CD, respectively. Accordingly, the peak for the amide I band shifted to 1510 cm^−1^ and the absorption peak for the amide II band was shifted to 1612 cm^−1^ [7]. The absorption band at 1345 cm^−1^ in the spectrum of poly(VPBA-AAm) microgel was attributed to the absorption peak of -B(OH)_2_ [44], which was also presented in the spectrum of poly(VPBA-AAm)-CD hybrid microgel, but the corresponding band shifted to 1350 cm^−1^.

The thermal stabilities of poly(VPBA-AAm)-CD and poly(VPBA-AAm) were investigated by thermogravimetric analysis (TGA). As demonstrated in Figure 2b, weight losses of 9.59% and 11.7% for poly(VPBA-AAm) microgel and poly(VPBA-AAm)-CD hybrid microgel, respectively, were observed in the temperature ranges of 25–320 °C and 25–380 °C, and this weight loss was mainly attributed to the loss of the physically adsorbed water molecules [45]. The skeleton decomposition of poly(VPBA-AAm)-CD was from 380 to 635 °C, and the weight loss at this stage was 57.7%. While the skeleton decomposition of poly(VPBA-AAm) was from 320 to 606 °C, the weight loss at this stage was 64.4%. After that, the weight of poly(VPBA-AAm)-CD and poly(VPBA-AAm) remained unchanged at 635 °C and 606 °C, respectively, and the residual weights were 30.56% and 25.97%, which were mainly boron oxides.

### 3.2. Fluorescence Properties of Poly(VPBA-AAm)-CD

**Figure 3:**
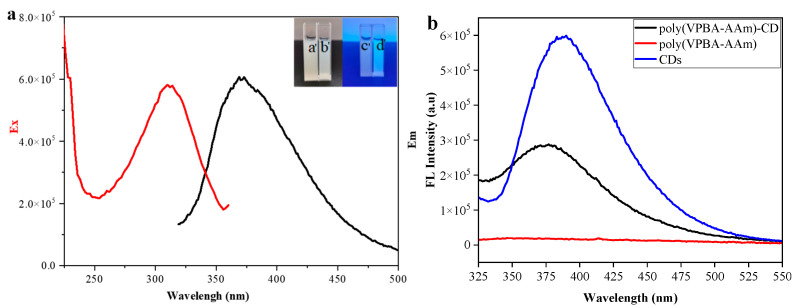
(**a**) Fluorescence spectra of poly(VPBA-AAm)-CD: the insets are the photographs of poly(VPBA-AAm)-CD and poly(VPBA-AAm) under natural light (**a**′,**b**′) and UV light at 365 nm (**c**′,**d**′). (**b**) Typical fluorescence spectra of free CDs, poly(VPBA-AAm) microgel and poly(VPBA-AAm)-CDs hybrid microgel, respectively (λ_ex_= 310 nm, 20 °C).

The fluorescence properties of the poly(VPBA-AAm)-CD hybrid microgel were investigated by dispersing the microgels in ultra-pure water. The optimal excitation and emission wavelengths for poly(VPBA-AAm)-CD in the fluorescence spectra shown in Figure 3a are 310 nm and 375 nm, respectively. The aqueous solutions of poly(VPBA-AAm)-CD emitted blue fluorescence under the radiation of a 360 nm UV lamp, while no fluorescence was observed in the poly(VPBA-AAm) solution. Both were milky white solutions under natural light. The optical properties of the poly(VPBA-AAm)-CD hybrid microgel, poly(VPBA-AAm) microgel and free CDs were also investigated and compared by testing their fluorescence spectra. As illustrated in Figure 3b, when the excitation wavelength was 310 nm, the maximum emission intensity of free CDs was located at 400 nm, while the poly(VPBA-AAm) microgel had no apparent emission peak. Compared with the fluorescence of free CDs, the poly(VPBA-AAm)-CD hybrid microgel had a typical wide emission peak similar to that of free CDs. However, the fluorescence intensity of the poly(VPBA-AAm)-CD hybrid microgel was obviously lower than that of CDs at a similar concentration and the emission peak had a blue shift, which proved that the CDs were successfully embedded in the inner microgel network of poly(VPBA-AAm). This result also proves that the fluorescence from the poly(VPBA-AAm)–CD hybrid microgels originating from the CDs was randomly complexed in the poly(VPBA-AAm) network.

**Figure 4:**
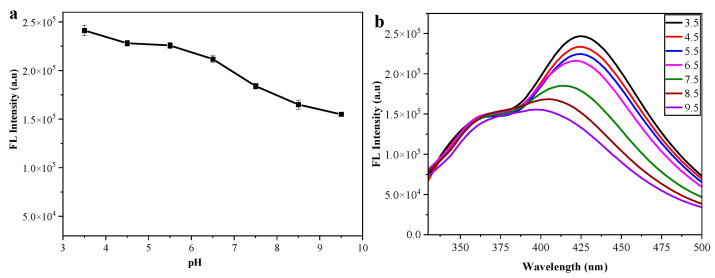
Influence of pH on the fluorescence intensity (**a**) and fluorescence emission spectrum (**b**) of poly(VPBA-AAm)-CD.

The fluorescence stability of the poly(VPBA-AAm)-CD solution at varying pH (3.5~9.5) was investigated. From Figure 4a, it can be concluded that the poly(VPBA-AAm)-CD solution has a relatively high fluorescence intensity in a wide pH range, and shows higher fluorescence in the acidic medium with a maximum fluorescence emission at pH 3.5. It can also be seen in Figure 4b that poly(VPBA-AAm)-CD exhibited different pH dependencies in its emission spectra. The fluorescence intensity of poly(VPBA-AAm)-CD at 410 nm reached the maximum at pH 3.5, and the emission spectra broadened and shifted towards blue with the increase in pH [46]. The results demonstrate that poly(VPBA-AAm)-CD has a pH-sensitive behavior.

### 3.3. Fluorescent Glucose Sensing of Poly(VPBA-AAm)-CD Hybrid Microgel

**Figure 5:**
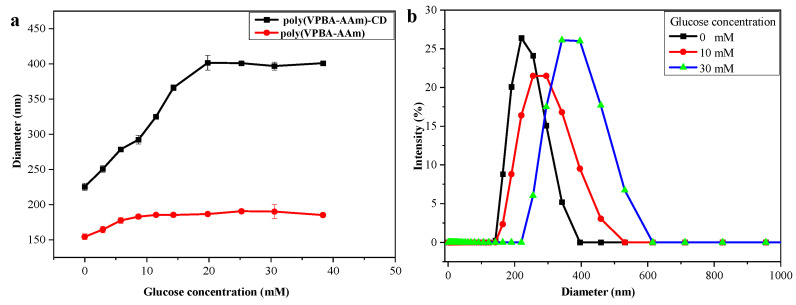
(**a**) Average Rh values (scattering angle θ = 90°) of poly(VPBA-AAm) and poly(VPBA-AAm)-CD at different glucose concentrations (PBS, pH = 7.4). (**b**) Size distribution of poly(VPB-AAm)-CD in PBS (pH = 7.4) with glucose concentrations of 0, 10 and 30 mM, respectively.

Figure 5a displays the glucose-responsive swelling curves of the poly(VPBA-AAm)-CD hybrid microgel at room temperature and at physiological pH. The curve represents the hydrodynamic radius (Rh) values of the hybrid microgels as a function of glucose concentrations. As shown in Figure 5a, the hydrodynamic sizes of poly(VPBA-AAm)-CD hybrid microgel and poly(VPBA-AAm) microgel gradually increased with the increase in glucose concentration. When the glucose concentration was higher than 20 mM, both microgels exhibited less obvious swelling changes with a further increase in glucose concentration. This was due to the limitation of chemical cross-linking, as the microgel had almost reached the maximal swelling ratio at a certain glucose concentration. Compared with the poly(VPBA-AAm) microgel prepared under the same conditions but without the addition of CDs, the hybrid microgel of poly(VPBA-AAm)-CD exhibited a larger size and higher swelling capacity, which was more conducive to the subsequent release of insulin loaded in the system at a high glucose concentration. Figure 5b illustrates the specific size distributions of the poly(VPBA-AAm)-CD hybrid microgel at glucose concentrations of 0, 10 and 30 mM, respectively. It can be seen that the size of the swollen microgels became increasingly non-uniform as the glucose concentration increased.

**Figure 6:**
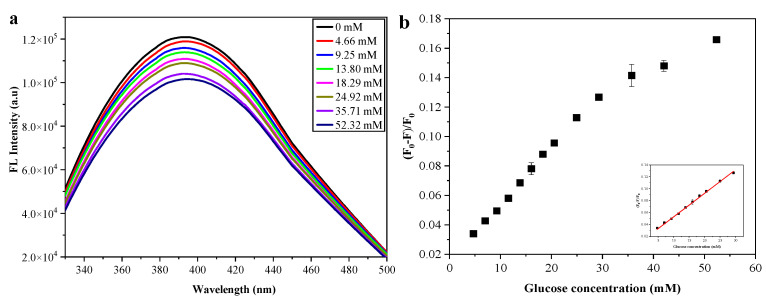
(**a**) Fluorescence spectra and (**b**) the variation of the fluorescence of poly(VPBA-AAm)-CD versus glucose concentration. The inset is the linear fitting plot of (F_0_ − F)/F_0_ versus glucose concentration (C_poly(VPBA-AAm)-CD_ = 1.75 mg/mL, PBS, pH = 7.4, λex = 310 nm).

Figure 6a displays the changes in the fluorescence spectra of the poly(VPBA-AAm)-CD hybrid microgel with increasing glucose concentrations at physiological pH. The results show that the fluorescence intensity of the poly(VPBA-AAm)-CD hybrid microgel continued to decrease with the gradual increase in glucose concentration in the solution until the glucose concentration reached 35.71 mM, and the declining fluorescence rate of the hybrid microgels slowed down. In order to explore the quantitative relationship between the fluorescence signal and glucose concentration, the curve of the fluorescence intensity (F_0_ − F)/F_0_ with the glucose concentration was plotted, where F_0_ and F were the fluorescence of the hybrid microgels before and after adding glucose in PBS, respectively. As illustrated in Figure 6b, the fluorescence intensity of the poly(VPBA-AAm)-CD hybrid microgel rapidly decayed as the glucose concentration increased from 0 to 29.28 mM, and then its fluorescence attenuation slowed down with the further increase in glucose concentration. Moreover, in the concentration range of 0–29.28 mM, the glucose-induced fluorescence intensity changes were linearly correlated with glucose concentrations (inset of Figure 6b), indicating that the hybrid microgel was promising as a glucose fluorescence sensor.

### 3.4. Glucose-Triggered Insulin Release of Poly(VPBA-AAm)-CD

**Figure 7:**
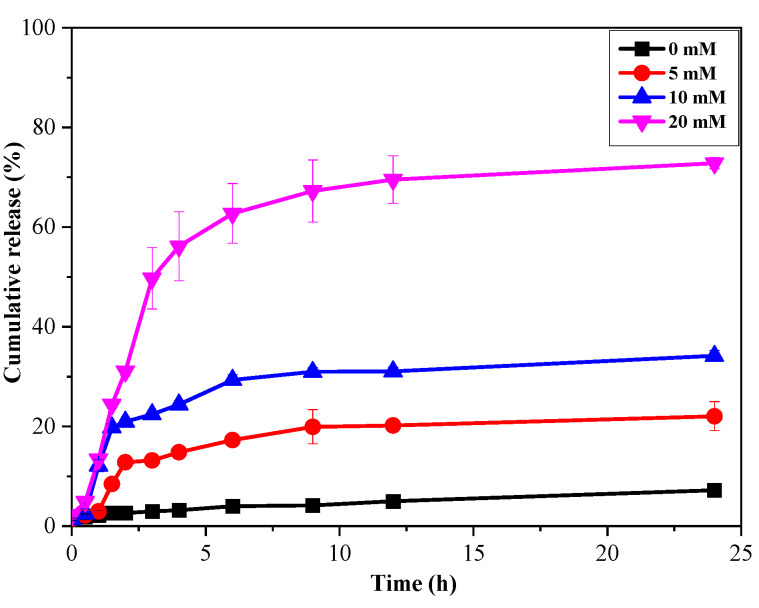
Insulin release profiles from hybrid microgels at different glucose concentrations (0 mM, 5 mM, 10 mM and 20 mM, 37 °C, pH = 7.4).

The hybrid microgel of poly(VPBA-AAm)-CD was further applied for glucose-regulated insulin delivery. There may be a certain host–guest hydrogen bond interaction between the amide/hydroxyl groups of hybrid microgels and insulin molecules, which gave the hybrid microgels a high insulin loading capacity [47]. The loading efficiency of insulin in the poly(VPBA-AAm)-CD hybrid microgel was calculated to be 15.1 ± 0.63%, and the encapsulation efficiency was 84.8 ± 4.4%. Figure 7 displays the in vitro release curves of insulin molecules from poly(VPBA-AAm)-CD dispersed in pH 7.4 PBS solution at 37 °C with different concentrations of glucose. Clearly, the increase in glucose concentration in the media can dramatically accelerate the release of insulin molecules from the hybrid microgels. Specifically, in the absence of glucose, only 8.1% of insulin molecules were released from the hybrid microgels within 24 h. In contrast, when the release medium contained a certain concentration of glucose (5 mM, 10 mM and 20 mM), the amount of insulin released from the hybrid microgels reached 25.1%, 35.7% and 74.6%, respectively, within 24 h.

### 3.5. Cytotoxicity of Poly(VPBA-AAm)-CD

**Figure 8:**
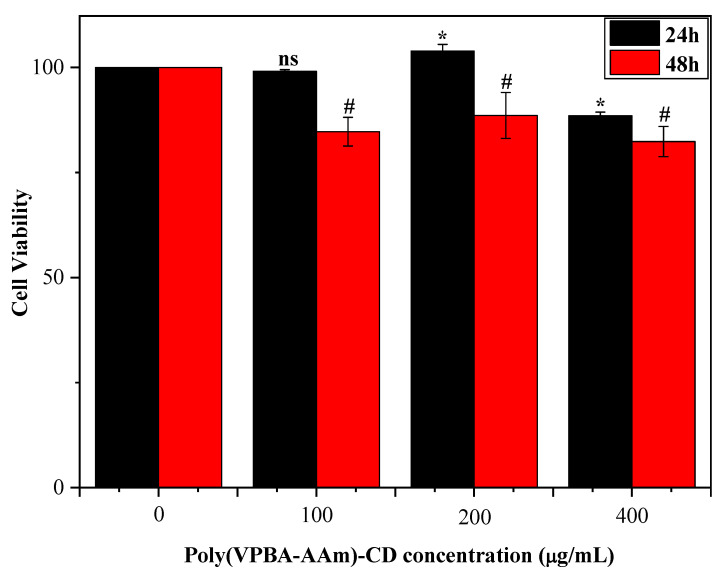
Viability of HL-7702 cells incubated with different concentrations of poly(VPBA-AAm)-CD for 24 h and 48 h by MTT assay. * *p <* 0.05 compared with the control group at 24 h, # *p* < 0.05 compared with the control group at 48 h, “ns” means no significance.

Because the high-glucose environment has a strong impact on hepatocytes, the liver is also a target organ of insulin resistance [48,49,50,51,52]. Hence, HL-7702 cells were applied to investigate the biocompatibility of hybrid microgels. Three different concentrations of poly(VPBA-AAm)-CD were tested for the cytotoxicity of HL-7702. The results in Figure 8 show that there was a significant difference between the tested groups and the normal group (*p* < 0.05). However, the cell viability results demonstrate that different concentrations of poly(VPBA-AAm)-CD had no obvious inhibition effect on the viability of HL-7702 cells after being co-cultured with cells for 48 h. As can be seen, when the concentration of poly(VPBA-AAm)-CD reached 400 µg/mL, the cell viability was still higher than 80% within 48 h, which confirmed the biological safety of the hybrid microgels. As we all know, acrylamide (AAm) monomer is a kind of chemical raw material which is widely used in the industry. Many studies have shown that acrylamide has significant neurotoxicity and is a recognized human carcinogen [53]. However, when it formed a polymer with biocompatible materials such as CDs, it had a low effect on the viability of HL-7702 cells at higher concentrations. However, the risks posed by polymer degradation in the body must also be considered. Therefore, in order to apply this material to a clinical diabetes treatment, it is necessary to comprehensively evaluate its safety from multiple aspects.

## 4. Discussion

Obviously, the poly(VPBA-AAm)-CD microgels were easily prepared by one-pot radical dispersion polymerization using the rationally designed hydrogen-bonded complex method. FTIR spectra, TGA results and fluorescence spectra confirmed the successful recombination of CDs with poly(VPBA-AAm). However, the hybrid microgels were not monodispersed. As evidenced by the SEM and TEM images in Figure 1, the poly(VPBA-AAm)-CD microgel had a wide size distribution which was mainly distributed between 100 and 400 nm. The non-uniform size of the hybrid microgels also leads to their non-monodispersity in the swollen state, as shown in Figure 5b. However, this did not affect the performance of the hybrid microgels, except for a signal delay when used for glucose detection [7]. This problem can be easily solved by detecting the fluorescence signal after a certain time incubation. Moreover, the peak shift in FTIR spectra indicated that there are strong hydrogen bonding interactions following CDs being embedded into the poly(VPBA-AAm) microgels. The different thermogravimetric loss results in Figure 2b also illustrate that the thermal stability of the hybrid microgel may be affected by the composite of CDs with poly(VPBA-AAm) [54], which reduced the weight loss of the poly(VPBA-AAm)-CD hybrid microgel in the second stage. After the CDs were embedded into the poly(VPBA-AAm) microgels, the final residual weight increased [55]. The results indicate that embedding the CDs increased the polymerization amount of 4-vinylphenyl boronic acid in the microgels, which was beneficial to improve the responsibility of the hybrid microgels to glucose. This result further indicates that there was an interaction (possibly through hydrogen bonding) between CDs and VPBA, which resulted in the increased amount of boron in the hybrid microgel.

The poly(VPBA-AAm) control microgels have no obvious emission peak. The poly(VPBA-AAm)-CD hybrid microgels demonstrated strong fluorescence properties with a characteristic emission peak similar to that of the free CDs, proving that the CDs with high fluorescence were successfully immobilized in the poly(VPBA-AAm) microgel system, which was mainly due to the stable hydrogen bonding complexation of the surface functional groups such as sulfhydryl/carboxyl/hydroxyl groups on CDs with the boronic acid groups of VPBA, the amide groups of AAm and the acyl group of EGDMA [56]. The fluorescence of the poly(VPBA-AAm)-CD hybrid microgels originated from the CDs complexed in the poly(VPBA-AAm) network. The blue shift may be due to the complexation of -OH and -COOH on the surface of CDs with amide/hydroxyl groups on the chain of a poly(VPBA-AAm) network. Since hydrogen bonding was affected by the pH of the media, the fluorescence intensity of the microgels was also affected. Figure 4 shows that the poly(VPBA-AAm)-CD solution had a relatively higher fluorescence intensity in the acidic medium. This could be explicated by the protonation and deprotonation of functional groups on the surface of CDs [57]. Because the amino, carboxyl, sulfhydryl and hydroxyl groups on the surface of CDs can easily form hydrogen bonds under alkaline conditions, this makes CDs aggregate and quench part of their own fluorescence, thus resulting in a decrease in the fluorescence intensity of the hybrid microgel. Under acidic conditions, these functional groups in CDs were apt for protonation, making them positively charged, and the CD molecules were more scattered due to the electrostatic repulsion, leading to a stronger fluorescence emission of the solution. In other words, due to the formation of hydrogen bonds under alkaline conditions, the electron cloud of the hybrid microgel would be more concentrated and the energy of the system would increase, resulting in a blue shift of the fluorescence peak. On the contrary, due to intermolecular repulsion, the electron cloud of the hybrid microgel became more dispersed, and the energy of the system decreased, resulting in a red shift of the fluorescence emission peak. The strong fluorescence of the microgel under acidic conditions is conducive to its development and application in oral administration.

Generally, the complexation of the cis-diols of glucose molecules with boric acids generated negatively charged boronates, which established Donnan potential and induced the swelling of the microgels [31,58]. The glucose-responsive transformation of the poly(VPBA-AAm)-CD network chain also changed the surface state of the CDs embedded in the network through hydrogen bonding interactions, thereby affecting the fluorescence characteristics of the hybrid microgels [7,56]. As such, the glucose-induced network swelling of poly(VPBA-AAm) hybrid microgels could suppress the fluorescence intensity of immobilized CDs. There may be two reasons that explain this. The first reason was that the swelling/de-swelling of the microgel network could change the original refractive index of the medium around CDs, thereby changing the Rayleigh scattering intensity [59]. The second reason was that the swelling/de-swelling of the microgel network could change the number of defects on the surface of CDs immobilized in the microgel network, which can change the fluorescence intensity of CDs by changing the partial charge density and spin state redistribution of CDs [60]. At higher glucose concentrations, microgel network chains tended to swell in the solution. Whereas hydrogen bonds between microgel chains and CDs hindered the expansion of the hybrid microgel to a higher swelling state, generating elastic tension in the hydrogen bonds at the microgel–CDs interface resulted in a surface state that could quench the fluorescence. The volumetric phase transition of the hybrid microgel in response to glucose can trigger insulin release. The increase in glucose concentration could cause the poly(VPBA-AAm)-CD microgel network to swell, which would unfold the pore diameter of the diffusion path and accelerate the release of loaded insulin molecules. The glucose-triggered insulin molecules release in the hybrid microgel is significant for maintaining blood glucose levels within the standard range, which opens up the possibility of achieving self-regulating insulin delivery on the basis of the body’s needs.

**Figure 9:**
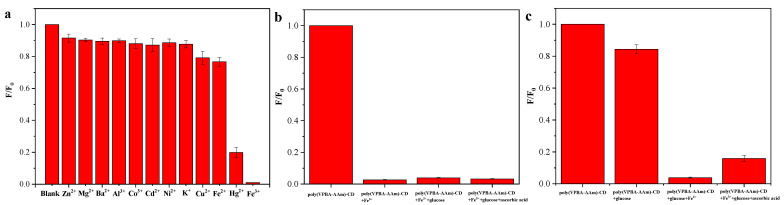
Influence of metal ions (**a**), Fe^3^^+^ (**b**) and glucose (**c**) addition in that order on the fluorescence intensity of poly(VPBA-AAm)-CD. Cpoly(VPBA-AAm)-CD = 0.26 mg/mL in pH 7.4 PBS, Cmetal ions = 3.0 mM, Cglucose = 10 mM, Cascorbic acid = 7.0 mM, λem = 375 nm, λex = 310 nm.

In order to further explore the mechanism of glucose response to the poly(VPBA-AAm)-CD hybrid microgel, the effects of different metal ions on the fluorescence intensity of the hybrid microgel were investigated based on our previous work [41]. As shown in Figure 9a, some metal ions such as Fe^2^^+^ and Cu^2^^+^ can slightly reduce the fluorescence intensity of poly(VPBA-AAm)-CD, while Hg^2^^+^ and Fe^3^^+^ have an obvious quenching effect on the fluorescence of poly(VPBA-AAm)-CD. The fluorescence intensity of poly(VPBA-AAm)-CD was 99% quenched by Fe^3^^+^. It was hypothesized that the high quenching efficiency of Fe^3^^+^ on poly(VPBA-AAm)-CD was due to the presence of abundant dihydroxyl groups (including boron hydroxyl groups) on its surface, which were good ligands for Fe^3^^+^. However, the coordination ability of the hydroxyl group with Fe^2^^+^ was much weaker than that with Fe^3^^+^ [61]. Therefore, Fe^3^^+^ has a strong quenching effect on the fluorescence of poly(VPBA-AAm)-CD, while Fe^2^^+^ has little effect on the fluorescence of poly(VPBA-AAm)-CD. According to this result, the hydrogen bond state or microenvironment in the microgel system was changed after the coordination of the hydroxyl groups and Fe^3^^+^. Due to the coordination of boron hydroxyl groups and Fe^3+^, the following added glucose would not be able to interact with the hybrid microgel, and the fluorescence of the system should not change significantly. Figure 9b confirms our speculation. According to our previous work, ascorbic acid can restore the quenched fluorescence of the CDs system. However, no fluorescence recovery was observed after adding ascorbic acid into the hybrid microgel system. This indicates that the complex formed by Fe^3+^ and the dihydroxyl groups on the hybrid microgels was stable, and Fe^3^^+^ cannot be reduced by ascorbic acid, so the fluorescence cannot be restored. It was also shown that VPBA was more susceptible to the Fe^3^^+^ reaction than glucose. Additionally, we switched the order of Fe^3^^+^ and glucose (Figure 9c). It was found that the fluorescence of the hybrid microgel system could be partially quenched when the concentration of glucose was 10 mM, and further increasing the concentration of glucose did not obviously change the fluorescence of the system. However, with the addition of Fe^3^^+^, the fluorescence of the system was almost completely quenched. Interestingly, when ascorbate was added to the system, the fluorescence of the system was partially restored. The reaction of VPBA with glucose was reversible, and the added Fe^3^^+^ first formed complexes with groups such as hydroxyl groups on the surface of CDs embedded in the microgels to quench the fluorescence. Since Fe^3^^+^ was more prone to the VPBA reaction than glucose, the excess Fe^3^^+^ competed with glucose to react with VPBA. During this process, Fe^3+^ was reduced to Fe^2^^+^ by ascorbic acid and the fluorescence of the system was partially recovered. This was an interesting phenomenon that deserves further exploration. However, the specific mechanism needs to be further proven by other techniques.

## 5. Conclusions

In conclusion, we successfully prepared a multifunctional and biocompatible hybrid microgel by a simple one-pot radical dispersion polymerization of the intellectually designed hydrogen-bonded complexes. The poly(VPBA-AAm)-CD microgel network was sensitive to glucose and can generate volume phase transitions according to the glucose concentration. Thus, the fluorescence intensity of the hybrid microgel can be changed by altering the physicochemical environment of the embedded CDs; thereby, the concentration change in glucose in the clinically relevant range can be sensed at a physiological pH. Thus, the insulin loaded into the poly(VPBA-AAm)-CD hybrid microgel can be triggered to be released by the concentration of glucose in the medium, which can further regulate the release of insulin according to the blood glucose level in diabetic patients.

## Data Availability

Not applicable here.
